# Predicted Infiltration for Sodic/Saline Soils from Reclaimed Coastal Areas: Sensitivity to Model Parameters

**DOI:** 10.1155/2014/317870

**Published:** 2014-08-13

**Authors:** Dongdong Liu, Dongli She, Shuang'en Yu, Guangcheng Shao, Dan Chen

**Affiliations:** Key Laboratory of Efficient Irrigation-Drainage and Agricultural Soil-Water Environment in Southern China, Ministry of Education, College of Water Conservancy and Hydropower Engineering, Hohai University, Nanjing 210098, China

## Abstract

This study was conducted to assess the influences of soil surface conditions and initial soil water content on water movement in unsaturated sodic soils of reclaimed coastal areas. Data was collected from column experiments in which two soils from a Chinese coastal area reclaimed in 2007 (Soil A, saline) and 1960 (Soil B, nonsaline) were used, with bulk densities of 1.4 or 1.5 g/cm^3^. A 1D-infiltration model was created using a finite difference method and its sensitivity to hydraulic related parameters was tested. The model well simulated the measured data. The results revealed that soil compaction notably affected the water retention of both soils. Model simulations showed that increasing the ponded water depth had little effect on the infiltration process, since the increases in cumulative infiltration and wetting front advancement rate were small. However, the wetting front advancement rate increased and the cumulative infiltration decreased to a greater extent when *θ*_0_ was increased. Soil physical quality was described better by the *S* parameter than by the saturated hydraulic conductivity since the latter was also affected by the physical chemical effects on clay swelling occurring in the presence of different levels of electrolytes in the soil solutions of the two soils.

## 1. Introduction

The term “infiltration” is used to describe the process of water soaking into, or being absorbed by, the soil [1]. Infiltration plays a pivotal role in the hydrologic cycle by effectively acting to partition water into surface and subsurface components [2]. Infiltration affects not only the water resources of plant communities but also the amount of overland flow and the associated processes of soil erosion and stream discharge [3]. Therefore, the predication of infiltration is an important issue in agricultural engineering, water resource management, and soil desalination.

Infiltration processes have been studied extensively over many years [4–10]. Infiltration within soils under given conditions is simultaneously affected by extrinsic factors (e.g., the seasonal distribution of precipitation, irrigation, soil compaction, etc.) and inherent soil heterogeneity (e.g., initial soil water content, soil texture, etc.).

Soil compaction affects bulk density, porosity, and pore shape and size distributions [6, 7]. The changes in these soil properties alter the soil water retention curve and hydraulic conductivity. In a review [8] of the effect of soil compaction on macroporosity and associated water movement found that it greatly affects water and solute flow, as well as plant root development. Furthermore, compaction increases the spatial variability of certain soil properties at the field scale while decreasing them at the mesoscale when compared with those of noncompacted soils. Zhang et al. [9] evaluated the effects of three levels of compaction on the hydraulic properties of two silty loam soils of the Loess Plateau in China. They reported that soil compaction significantly altered the soil water retention curves and influenced other hydraulic properties in various ways; for example, one of the soils exhibited significant decreases in saturated hydraulic conductivity with increasing compaction whereas unsaturated hydraulic conductivity did not significantly change in the measured water volume ratio range.

The soil water potential due to the ponded water depth (*ψ*_top_) also has an effect on the infiltration process. Philip [4] showed that the infiltration rate and cumulative infiltration both increase with increasing *ψ*_top_. Chowdary et al. [11] arrived at a similar conclusion and reported that increases in *ψ*_top_ resulted in increases in cumulative infiltration.

Another research has focused on the effects of inherent soil heterogeneity on the infiltration processes. Studying the effect of initial soil water content *θ*_0_ on infiltration, Wangemann et al. [5] found that infiltration rates after one hour of water application on soils having an *θ*_0_ value of more than 15% were greater than in the same soil where *θ*_0_ was less than 5%; this was ascribed to the greater aggregate stability of the wetter soils. In addition, Shen et al. [10] noted that under irrigation the size of the wetted zone in the soil profile increases with increasing *θ*_0_.

Coastal areas in China have a long history of reclamation from the sea. In the reclaimed soils, various compaction conditions (e.g., wetting and drying cycles, compaction by vehicular traffic, cultivation, drainage, etc.) have clearly increased the spatial variability of soil bulk density. In addition, such soils with low stability of structure disperse and slake when wetted by irrigation water, and the pores would be filled with fine particles. Thus, a crust may be developed as the soil surface dries. This crust presents a barrier for infiltration. Knowledge of the infiltration processes in these reclaimed areas is a prerequisite to understanding the mode of water supply and soil desalination. It is necessary to identify the factors that contribute to the infiltration processes. However, there is no available information about the effects of soil surface conditions and *θ*_0_ on water movement in the unsaturated soils in these reclaimed coastal areas.

The objectives of this study are (1) to develop a model to describe soil water infiltration processes in the sodic/saline soils of reclaimed coastal land and (2) to examine and discuss the effects of compaction, ponded water depth (*ψ*_top_), and the initial soil water content (*θ*_0_) on water retention characteristics, hydraulic conductivity, and other infiltration processes in these soils.

## 2. Materials and Methods

### 2.1. Infiltration Experiment

**Figure 1:**
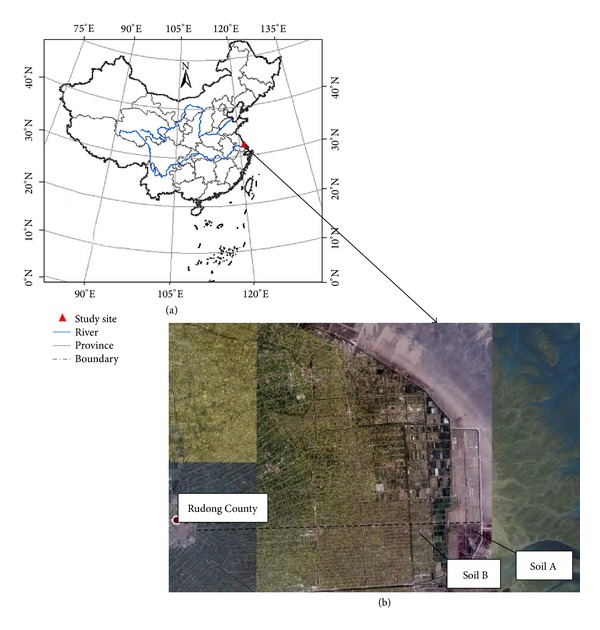
Location of the study site in Jiangsu Province, China (a) and of the two soil collection sites that were reclaimed in different years (b) (Soil A: 2007; Soil B: 1960).

A series of infiltration experiments were conducted on soils packed into transparent acrylic columns (5 cm inner diameter, 40 cm length). The soils were collected from two locations in Rudong County, Nantong City (from 120°42′ to 121°22′ E and from 32°12′ to 32°36′ N), Jiangsu province, China (Figure 1(a)). Land reclamation from the sea began in Rudong County as early as the 20th century, and this process has accelerated in the past 60 years. The two sodic soils were designated as Soil A and Soil B (Figure 1(b)), and their properties and some details of the sampled sites are presented in Table 1. Soil particle size was analyzed using a MasterSizer2000 laser particle size analyzer (Malvern Instruments, UK). Soil organic matter was determined by the Walkley-Black method [12]. Soil electrical conductivity of a 1 : 5 soil (EC) to water extract was determined using a DDS-307 conductivity meter (Shanghai Precision Scientific Instrument Co. Ltd., Shanghai). Exchangeable cations were extracted by ammonium acetate [13], and sodium was determined by a flame photometer [13]. The exchangeable sodium percentage (ESP) was calculated; ESP = 100 × *C*_Na_/CEC, where *C*_Na_ denotes the concentration of the exchangeable Na^+^ (cmol_c_/kg) and CEC is the cation exchange capacity (cmol_c_/kg) [13]. Saline is used in connection with soils for which EC > 4 mS/cm and ESP > 15. Saline-sodic is applied to soils for which EC > 4 mS/cm and ESP > 15. Nonsaline-sodic is applied to soils for which ESP > 15 and EC < 4 mS/cm [14]. While both soils were sodic, having an ESP greater than 15, only Soil A was considered to be saline based on the electrical conductivity of the soil : water solution. Disturbed soil samples from the top 20 cm soil layer were collected, air dried, sieved through a 2 mm screen, and thoroughly mixed. The gravimetric water content of the air-dried soils was determined by measuring the loss in mass during drying to constant mass in an oven at 105°C. The air-dried soils were uniformly packed into the columns to achieve the desired bulk densities, that is, 1.4 g/cm^3^ or 1.5 g/cm^3^ for both soils. This was achieved by packing a soil layer of 5 cm thickness to the required bulk density before packing the next soil layer. Seven soil layers were packed into each of the columns. The surface of each soil layer was roughened before adding the next layer in order to reduce discontinuities between the layers. The upper soil layer was protected from the effects of drop impact and inflowing water by a circle of filter paper.

**Figure 2:**
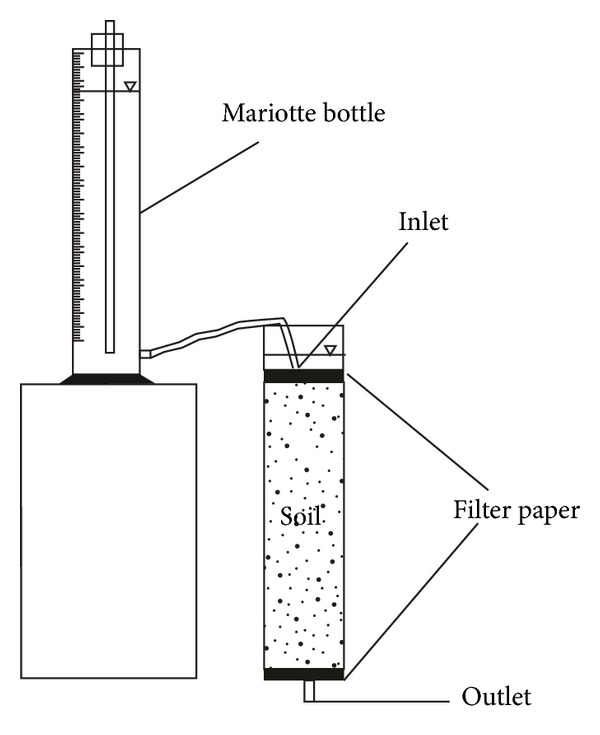
Schematic diagram of the infiltration experiment setup.

A Mariotte bottle was used to maintain a constant ponded water depth on the top of each soil column. A tube connected the Mariotte bottle to a fixed height (ranging between 0 and 5 cm) above the soil surface. A schematic diagram of the infiltration experiment setup is shown in Figure 2. Soil hydraulic parameters and experimental conditions are presented in Table 2.

The infiltration experiments proceeded until the wetting front reached the bottom of the soil column. During these experiments, the cumulative infiltration and infiltration rate were determined from the changes in the water level in the Mariotte bottle measured as a function of time. The depth of the wetting front was also measured to determine the mean rate of the water flow in the unsaturated soil. After each experiment ended, the gravimetric soil water content of soil samples taken from seven locations within the soil column was measured by loss of mass during oven drying at 105°C. The results were to compare with both the results simulated by the model based on Richard's equation and the results of the Hydrus-1D model simulation. In this study, the effect of evaporation on the infiltration process was not considered and, in this set of experiments, evaporation would have been negligible.

### 2.2. Hydraulic Properties of the Soil Profile

The soil water content (*θ*) was calculated by the following equation:

(1) $$\begin{array}{c} \theta =\frac{\omega \rho_{b}}{\rho_{w}}, \end{array}$$

where *ω* is the gravimetric soil water content (g/cm^3^), *ρ*_*b*_ is dry bulk density (g/cm^3^), and *ρ*_*w*_ is density of water (1 g/cm^3^ at standard temperature and pressure).

Pairs of data points for the soil water content (*θ*) and the soil water potential (*ψ*), that is, (*θ*, *ψ*), were obtained for both soils using the pressure plate method [9]. The values of *ψ* were set as 0, 1, 10, 20, 50, 80, 200, 300, 500, 1000, 3000, and 15000 cm. These points were used to fit the van Genuchten equation [15] to obtain soil water retention curves (*θ*(*ψ*)) for each soil. The hydraulic properties (*K*(*ψ*) and *θ*(*ψ*)) were calculated by the van Genuchten equation:

(2) θ(ψ)={(θsat⁡−θres)[1+|αψ|n]m+θres,ψ<0,θsat⁡,ψ>0,Se=(θ−θres)(θsat⁡−θres),K(ψ)=KsSe1/2[1−(1−Se1/m)m]2,

where *ψ* is the soil water potential, that is, equivalent to the pressure head in these experiments (cm), which is positive under saturated conditions and negative under unsaturated conditions; *θ*, *θ*_res_, and *θ*_sat⁡_ are the measured, residual, and saturated soil water contents (cm^3^/cm^3^); *α* (1/cm) and *n* (dimensionless) are unsaturated soil parameters related to the inverse of the air entry suction and the pore size distribution, respectively; *m* = 1 − 1/*n*; *S*_*e*_ is the effective saturation; and *K*(*ψ*) and *K*_*s*_ are the unsaturated and saturated hydraulic conductivities. The corresponding soil hydraulic parameters of each soil are given in Table 2.

### 2.3. The Soil Physical Quality Index

A soil physical quality index is useful for assessing the outcomes of management practices on the soil for different land use patterns. Dexter [16] defined a soil physical parameter (*S*) that was equal to the slope of the soil water retention curve at its inflection point and that could be used as an index of soil physical quality. Therefore, *S* was used in this study as a soil physical quality index that could determine the influences of long-term reclamation in the coastal region. The curve of soil water content against log *ψ* has only one characteristic point, which is the inflection point [16], the value of the slope *S* at the inflection point (*θ*_*i*_, *ψ*_*i*_), and the location of the point could be calculated from the parameters of the van Genuchten equation for the soil water retention curves:

(3) θi=(θsat⁡−θres)[1+1m]−m+θres,ψi=−1α[1m]1/n,S=−n(θsat⁡−θres)[2n−1n−1](1/n−2).

The *S* values of each soil are shown in Table 2.

## 3. Simulation of Hydraulic Parameters Related to Infiltration Based on Richard's Equation

For one-dimensional vertical flow in an unsaturated soil, Richard's equation [17] is given as follows:

(4) $$\begin{array}{c} C\left(\psi \right)\frac{\partial \psi }{\partial t}=\frac{\partial }{\partial z}\left(K\left(\psi \right)\frac{\partial \psi }{\partial z}\right)-\frac{\partial }{\partial z}K\left(\psi \right), \end{array}$$

where *C*(*ψ*) is the specific moisture capacity (1/cm) [3], *t* is time (min), and *z* is the vertical coordinate, which is positive in the downward direction.

Boundary conditions in the column infiltration experiments included a constant-head (*ψ*_top_ > 0) at the upper end and free drainage at the lower end (*z* = *L*); the initial conditions and upper boundary conditions were as follows:

(5) $$\begin{array}{c} t=0,\;\psi =\psi_{\text{st}},\;0\leq z\leq L,\\ t>0,\;\psi =\psi_{\text{top}},\;z=0,\\ t>0,\;\frac{\partial \psi }{\partial z}=0,\;z=L, \end{array}$$

where *ψ*_st_ is the initial potential water (cm) and *L* is the distance from the soil surface to the bottom of soil column (cm).

An implicit finite difference method was used to solve Richard's equation. The differential format was as follows:

(6) $$\begin{array}{c} a_{i}\psi_{i-1}^{j+1,k+1}+b_{i}\psi_{i}^{j+1,k+1}+c_{i}\psi_{i+1}^{j+1,k+1}=f_{i}. \end{array}$$

The coefficients (*a*_*i*_, *b*_*i*_, *c*_*i*_, and *f*_*i*_) were calculated using the following equations [18]:

(7) ai=−Kij+1,k+Ki−1j+1,k2Δz,bi=Kij+1,k+Ki+1j+1,k2Δz+Kij+1,k+Ki−1j+1,k2Δz+ΔzΔtCij+1,k,ci=−Kij+1,k+Ki+1j+1,k2Δz,fi=ΔzΔtCij+1,kψij+1,k−ΔzΔt(θij+1−θij)−Ki+1j+1,k−Ki−1j+1,k2,

where Δ*t* and Δ*z* are the time and space intervals, *i* and *j* are the space and time levels, and *k* is the iteration time at a given time. In this study, a self-written Matlab program was used to solve these equations. For all modeled cases, *L* was fixed at a large enough value so as to be unaffected by the wetting front, thus ensuring that the lower boundary condition was valid. More details [19] about the values of *L*, Δ*t*, and Δ*z* were shown.

In order to assess the applicability of this model, we made the comparisons of values simulated by this model and those obtained by either measurement or from the Hydrus-1D model [20]. The statistical approach involved the calculation of the normalized root mean square error (RMSE). Consider the following:

(8) $$\begin{array}{c} \text{RMSE}=\sqrt{\frac{1}{N}\overset{N}{\underset{i=1}{\sum }}{\left(s_{i}-m_{i}\right)}^{2}}, \end{array}$$

where *N* is the number of data points (*N* = 7) and *s*_*i*_ and *m*_*i*_ are the simulated and measured values of the *i*th point, respectively.

However, the purpose of setting up this model was not to construct a new infiltration formula but to assess the applicability of the existing ones for the sodic/saline soils from reclaimed coastal areas.

## 4. Results and Discussion

### 4.1. Model Validation and Sensitivity Analysis

**Figure 3:**
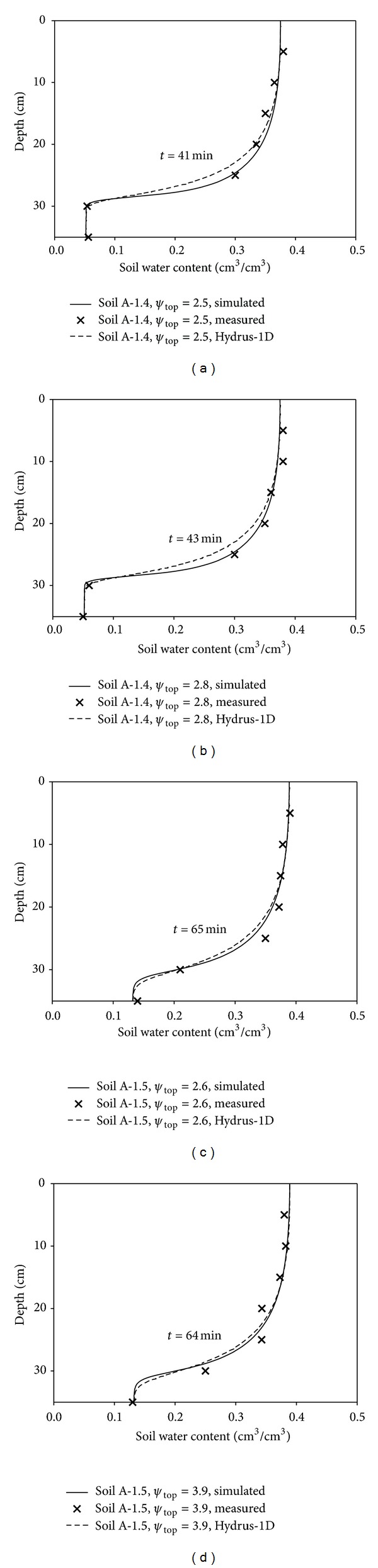
Comparison of the model simulations of the soil water retention curve with measured data and the Hydrus-1D simulation for columns of Soil A packed to two different bulk densities (1.4 or 1.5 g/cm^3^) for various ponded depths (*ψ*_top_) and infiltration times (*t*).

Figure 3 enables visual comparisons to be made of the soil water content values within the soil profile that were simulated by the model based on Richard's equation with both the measured data and the results of the Hydrus-1D model simulation. RMSE was used to quantify the deviations between values simulated by the model and those obtained by either measurement or from the Hydrus-1D model. All of the RMSE values were about 1% (data not shown). This indicates that the model simulated the measured data very well and that it can be used to simulate the infiltration process in the two sodic soils, whether saline or not. It further indicates that the model simulations were comparable with those of Hydrus-1D.

A sensitivity analysis of the parameters *α* and *n* in the van Genuchten equation should be made. The parameter *α* can describe the capillary fringe thickness and is related to the inverse of the air entry value, which is assumed to be related to the maximum pore size [21]. The parameter *n* can reflect the pore size distribution of the soil and is related to the pore size distribution index (*λ*) by *n* = *λ* + 1 [22].

**Figure 4:**
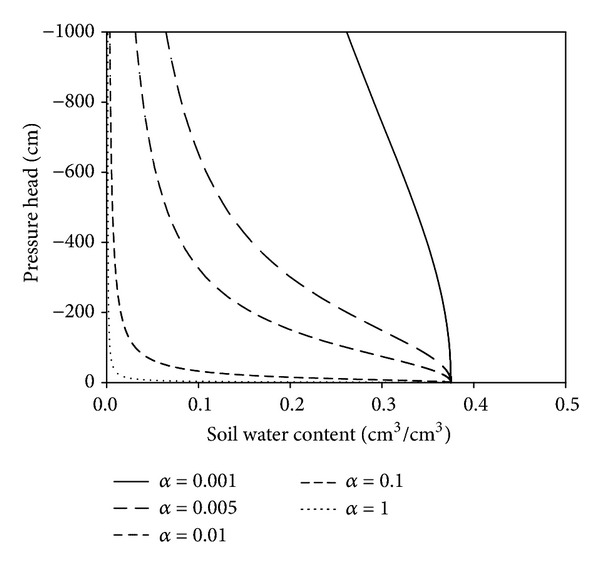
Relations between pressure head and soil water content for five different values of the *α* parameter used in the simulation model.

**Figure 5:**
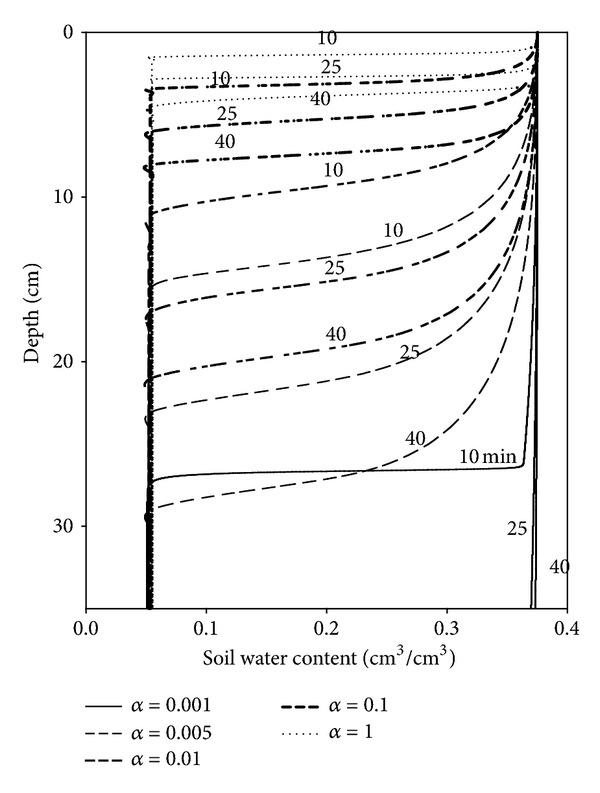
Simulated soil water content profiles after different infiltration times (10, 25, and 40 min) for different values of the *α* parameter in the model.

**Figure 6:**
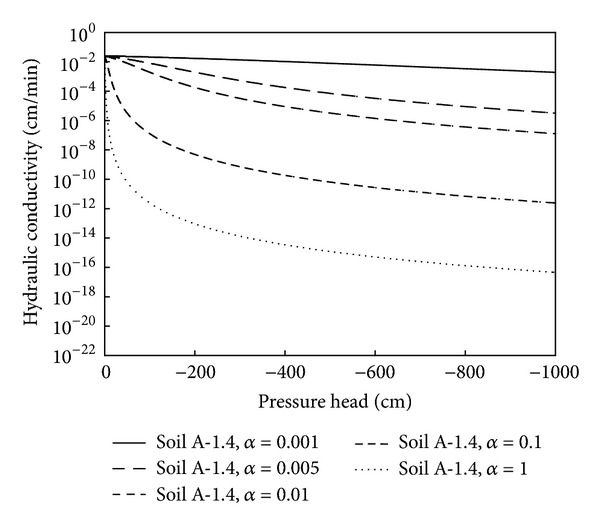
The relation between pressure head and hydraulic conductivity for five different values of the *α* parameter used in the simulation model.

The effect of parameter *α* on the simulated water retention curve and other hydraulic properties of Soil A having a bulk density of 1.4 g/cm^3^ was examined by changing the *α* values in the range from 0.001 cm^−1^ to 1 cm^−1^, using these five values: 0.001, 0.005, 0.01, 0.1, and 1 cm^−1^. At the same time, the other parameters (*n*, *K*_*s*_, *θ*_*s*_, and *θ*_*r*_) were kept constant and *ψ*_top_ was set at 2.5 cm. Lower *α* values resulted in the simulation predicting greater water retention in Soil A for a given pressure head (Figure 4). Soils with lower *α* values may have smaller capillary forces and may be more likely to be affected by gravity drainage. Increasing the value of *α* resulted in reductions in the distance and rate of the wetting front advancement (Figure 5). For instance, when *α* was 0.1 and the infiltration time increased from 10 to 25 min, the wetting front advanced 2.4 cm from a depth from 3.6 to 6.0 cm. However, when *α* was increased to 1, the wetting front advanced only 1.4 cm from a depth from 1.5 to 2.9 cm for the same infiltration time interval. In fact, as *α* increased and the texture class varied from clay to sand, the *K*_*s*_ often show an increasing trend [23]. Thus, this may be expected because, as shown in Figure 6, the hydraulic conductivity (*K*) increased when *α* decreased for a given pressure head, which would lead to a faster advancement of the wetting front. However, as the soil wets and the pressure head approaches zero, the effect of *α* is reduced. It should be noted that *α* had a notable and significant effect on *K* and that *K* itself had an important role in the infiltration process.

**Figure 7:**
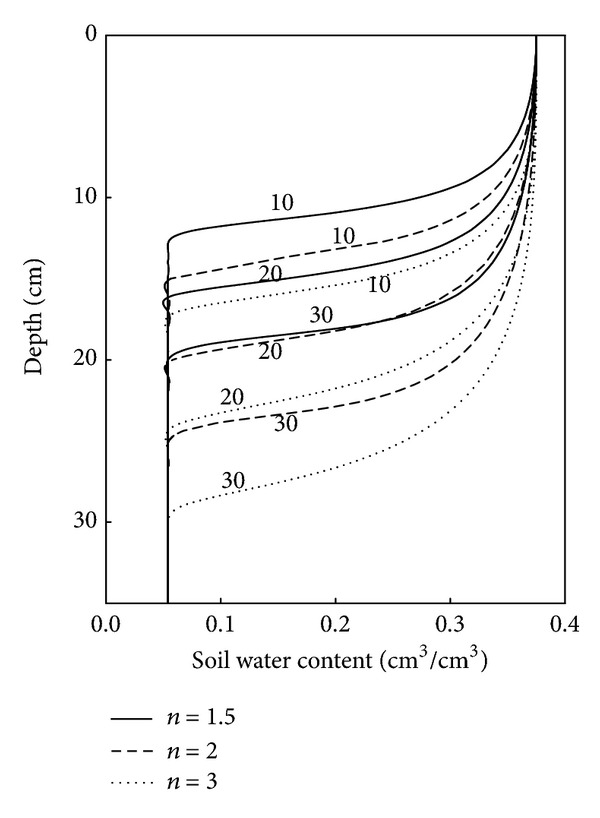
Simulated soil water content profiles after different infiltration times (10, 20, and 30 min) for different values of the *n* parameter in the model.

Similar to the examination of *α*, the effect of parameter *n* on the simulated water retention curve and other hydraulic properties of Soil A having a bulk density of 1.4 g/cm^3^ was studied by varying *n* in the range from 1.5 to 3, using the three values of 1.5, 2, and 3. As before, other parameters (*α*, *K*_*s*_, *θ*_*s*_, and *θ*_*r*_) for the soil remained unchanged and *ψ*_top_ was fixed at 2.5 cm.  Figure 7 shows the depth of the wetting front at three different times (10, 20, and 30 mins) after the commencement of infiltration for the three *n* values. The effect of increasing *n* resulted in the wetting front moving at a greater rate (Figure 7) and in increases in cumulative infiltration (data not shown). For instance, when *n* was 1.5, the wetting front had advanced to depths of 12, 16, and 20 cm for infiltration times of 10, 20, and 30 mins, respectively, while the cumulative infiltration was 34.3, 45.2, and 56.0 mm, respectively. When the value of *n* was increased to 2, the corresponding depths of the wetting fronts increased to 15, 21, and 25 cm while the cumulative infiltration increased to 41.3, 56.1, and 70.0 mm. This phenomenon may be due to wider pore size distributions and the relative ratio of the smaller pores, which enhanced the infiltration process, as *n* decreased. Similarly, Dexter [16] showed that *n* decreased as FAO/USA textural class changed from a sandy to a clay soil. In addition, increases in *n* resulted in increases in *S* (Table 2), which indicated that soils with a higher value of *n* that have wider distributions in pore size might have a better physical quality.

### 4.2. Effect of Compaction and Ponded Water Depth on Water Movement

**Figure 8:**
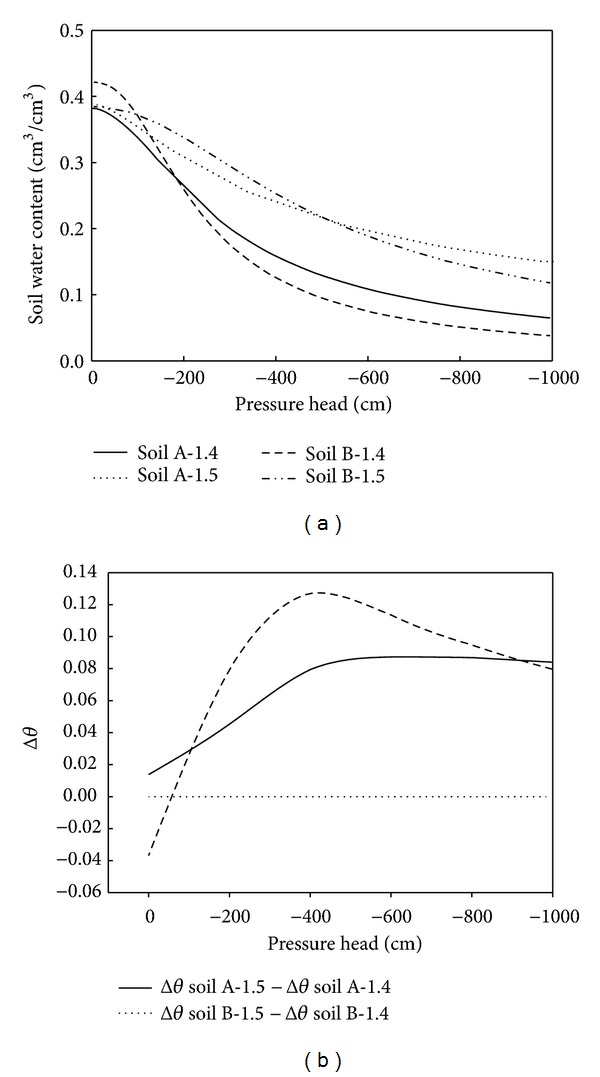
(a) Simulated soil water retention curves for two soils columns packed at different bulk densities (1.4 or 1.5 g/cm^3^); (b) the differences in soil water content (Δ*θ*) at the same pressure head.

Compaction reduced both the volume of a given mass of soil and the volume of the soil pores, which affected the soil water retention curve (Figure 8(a)).  Figure 8(b) presents, for each soil, the differences in the *θ* (Δ*θ*) between the two studied bulk densities (1.4 and 1.5 g/cm^3^) when the pressure head was the same; Δ*θ* = *θ*_*i*_ − *θ*_*j*_, where *i* and *j* denote the given soils with bulk densities of 1.5 g/cm^3^ and 1.4 g/cm^3^, respectively. For Soil A, Δ*θ* increased gradually as the pressure head approached zero attaining the maximum value (0.08 cm^3^/cm^3^) when the pressure head was −600 cm, after which it decreased sharply to a value of 0.02 cm^3^/cm^3^ when the soils were saturated. The effect of compaction on the volumetric soil water content was to increase it across the measured tension range. For Soil B, the changes in Δ were greater than those observed for Soil A and the maximum value (0.13 cm^3^/cm^3^) occurred when the pressure head was −400 cm. The maximum Δ thus occurred when Soil B was closer to saturation than when Soil A was. Furthermore, in contrast to Soil A, compaction resulted in small decreases in the volumetric soil water content (0 to 0.04 cm^3^/cm^3^) when the pressure head was between −100 and 0 cm. These results indicated that the compaction caused notable changes in the water retained by Soil A and by Soil B and that generally these changes resulted in increases of volumetric soil water content for both soils but, in the case of Soil B, there was a small decrease when the soil was close to saturation. There was a difference between the two soils, which would be related to the structure and aggregate stability of the soils. Soil B should have a better structure since it has been reclaimed for longer allowing more time for structural development to occur; and because it has a lower ESP, it was relatively less susceptible to clay dispersion. Even so, both soils were still very prone to dispersion in the presence of deionized water. However, the larger amount of free salts in Soil A may mitigate the dispersion. Thus, a better structure of Soil B also meant that it could be destroyed to a greater extent by compaction resulting in generally larger Δ*θ* values for Soil B than for Soil A.

Compaction can affect the capillary fringe thickness, pore size distribution, and hydraulic conductivity. The values of parameter *α* decreased with increasing compaction for both soils (Table 2), indicating increases in air entry tension. The values of parameter *n* decreased as the compaction increased, indicating that compaction had a negative effect on the soil pore size distribution.

Compaction also caused notable decreases in *K*_*s*_ values (by 20% for Soil A and by 50% for Soil B) but not to the same extent as its effects on *α* and *n* (Table 2). Similar findings [9, 24] were reported. Furthermore, there was a considerable difference in *K*_*s*_ between Soil A and Soil B at the same compaction level. This may indicate that long-term reclamation greatly and adversely affected the saturated hydraulic conductivity. Both soils were sodic and, if the soil solution contained low levels of electrolytes, would be prone to clay swelling, which would have blocked water-conducting pores [25]. However, Soil A was also a saline soil and the high level of soluble salts probably reduced the swelling leading to its higher *K*_*s*_ value (Table 2). In this respect, the reduction in salinity due to leaching following during 53 years of reclamation was detrimental to the saturated hydraulic conductivity of the soil, although other activities such as cultivation should have a beneficial effect on soil structure. In time, the ESP of Soil B should be further reduced by appropriate land management practices, which may include adding calcium compounds such as lime or gypsum, so that the adverse effect of clay swelling should be reduced. In this study, the *K*_*s*_ of Soil B was more sensitive to compaction than that of Soil A.

Saturated hydraulic conductivity *K*_*s*_ has often been used to evaluate the effect of compaction on water movement since *K*_*s*_ values are governed by the abundance of relatively large pores and their continuity [7, 8]. However, as postulated above, in sodic soils with different salinities this parameter may not reflect the physical quality of the soil in terms of its structure due to the effect of clay dispersion. Clay dispersion causes clay particles to fill the soil pores, leading to a reduction in soil permeability and loss of soil structure [26]. Thus, the hydraulic conductivity is also reduced. On the other hand, the clay contents are relatively low for Soil A and Soil B (9 and 11%). This will limit the effects of the clay dispersion on the hydraulic properties, but this depends on the particle size distribution of the soils. The structural quality of the soil directly affects water retention. Therefore, the *S* parameter of the water retention curve may better reflect the effect of long-term reclamation on soil physical quality. From Table 2, the value of *S* was greater for Soil B than for Soil A and decreased by 31% and 23% for Soil A and Soil B, respectively, with the increase in compaction. Compaction notably affected the water retention in Soil A and Soil B. Zhang et al. [9] similarly observed that the water retention of Mizhi soil (a Calcic Cambisol) was significantly influenced by compaction across a wider tension range than Heyang soil (a Chromic Cambisol), and that there was a significant difference between the two compaction treatments (10% and 20%). However, Green et al. [24] found that compaction did not significantly affect water retention in several soils in different landscapes; for example, there were little or no compaction effects on water retention for the Akron site (a silt loam soil) averaged separately over wheel-track and no-track areas. The mean soil bulk densities for wheel-track and no-track cores were 1.45 ± 0.07 and 1.39 ± 0.07 g/cm^3^. These different findings suggest that the effect of compaction depends on the soil types as well as upon the degree of compaction. Clearly compaction had a notable effect on the physical quality of the soils in this study as indicated by the *S* value. Similarly, Dexter [16] found that the value of *S* significantly decreased with increasing bulk density (from 1.35 to 1.65 g/cm^3^) in a sandy clay loam Spanish soil. Therefore, the compaction may be responsible for the observed changes in saturated hydraulic conductivity and soil physical quality for Soil A and Soil B but the former was also affected to a greater extent by soil sodicity and soil salinity. For Soil A, The primary physical process induced by high sodium concentrations (ESP > 15) is soil dispersion while soil water salinity (EC > 4 mS/cm) can affect soil physical properties by causing flocculation.

**Figure 9:**
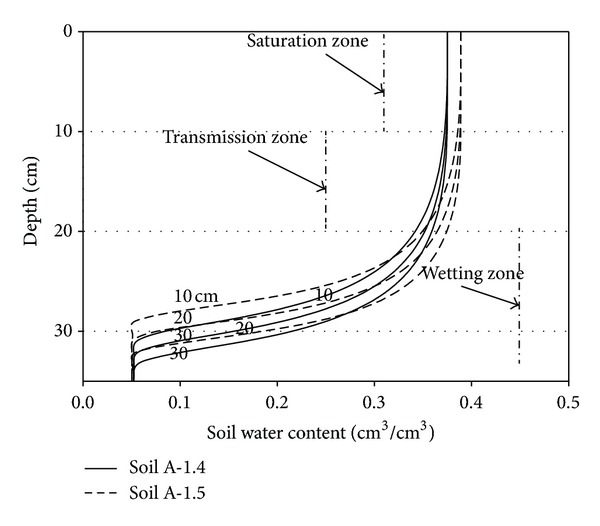
Simulated soil water content profiles after 30 min of infiltration in columns of Soil A with two different bulk densities (1.4 or 1.5 g/cm^3^) for different ponding depths (10, 20, and 30 cm).

When a layer of water is rapidly applied to, and thereafter maintained on, the surface of a soil, the soil infiltration process can be affected by the ponded water depth. To investigate the influence of ponded water depth (*ψ*_top_) on the infiltration process, a simulation study using the model created for this study was carried out using three different values of *ψ*_top_ for Soil A with two different bulk densities (1.4 and 1.5 g/cm^3^). The values of *ψ*_top_ used were 10, 20, and 30 cm, and the value of *θ*_0_ was fixed at 0.05 cm^3^/cm^3^. The duration of the infiltration simulation was 30 min. The cumulative infiltration and the wetting front advancement rate both increased with increasing ponded water depth (Figure 8). From Figure 9 it can be seen that the wetting front depths were 31, 32, and 34 cm when the *ψ*_top_ values were 10, 20, and 30 cm, respectively, for Soil A with a bulk density of 1.4 g/cm^3^. However, when Soil A was compacted to a bulk density of 1.5 g/cm^3^, the corresponding wetting front depths were only 28, 30, and 31 cm. These results indicate that the wetting front depth was affected by compaction and decreased as the compaction increased. Furthermore, for the three ponded depths the cumulative infiltration amounts were 86.5, 91.0, and 95.2 mm, respectively, for Soil A with a bulk density of 1.4 g/cm^3^, and were 85.7, 91.9, and 97.5 mm when the bulk density was 1.5 g/cm^3^. These results show that cumulative infiltration decreased slightly with an increase in compaction when *ψ*_top_ = 10 cm while it increased with compaction for *ψ*_top_ ≥ 10 cm. However, the influence of ponded water depth on the infiltration process appears to be limited. For the changes in ponded depths between 10 and 20 cm and between 20 and 30 cm, and when the bulk density of Soil A was 1.4 g/cm^3^, the wetting front depth only increased by 3.2% and 6.0%, respectively, and the cumulative infiltration only increased by 5.1% and 4.6% whereas the corresponding increases in the ponded depths were 100% and 50%. When the bulk density was 1.5 g/cm^3^, the magnitude of the effect of the ponding depth on the wetting front depth (4.1% and 5.5%) and the cumulative infiltration (5.7% and 4.0%) was similar.

When a layer of water is applied to the soil surface rapidly, the presence of the water now exerts a pressure on the soil surface that would be significantly greater than the atmospheric pressure. The delivery rate exceeds the soil's infiltration capacity, and thus the infiltration process becomes soil-controlled. In this study, Soil A's water content profile during infiltration can be divided into three parts: a saturation zone, a transmission zone, and a wetting zone. From Figure 9, it can be seen that the thicknesses of the saturation and transmission zones for the all ponded water depths were generally equal. Furthermore, the depths of the wetting zones were similar for the various ponded water depths. Therefore, although the hydraulic conductivity and the hydraulic gradient of the wetting zone were affected by the ponded water depth, the degree to which they were affected was not remarkable. Consequently, increasing the ponded water depth would not benefit the process of soil desalination by leaching.

### 4.3. Effects of Initial Soil Water Content on the Infiltration Process

The initial water content can exert a great effect on infiltration [27]. In order to investigate the effect of *θ*_0_ on the infiltration process, simulations were carried out in which *θ*_0_ was fixed at values of 0.05, 0.1, or 0.15 cm^3^/cm^3^. The ponded water depth was kept constant at 2.5 cm and the duration of the infiltration simulation was 30 min.

**Figure 10:**
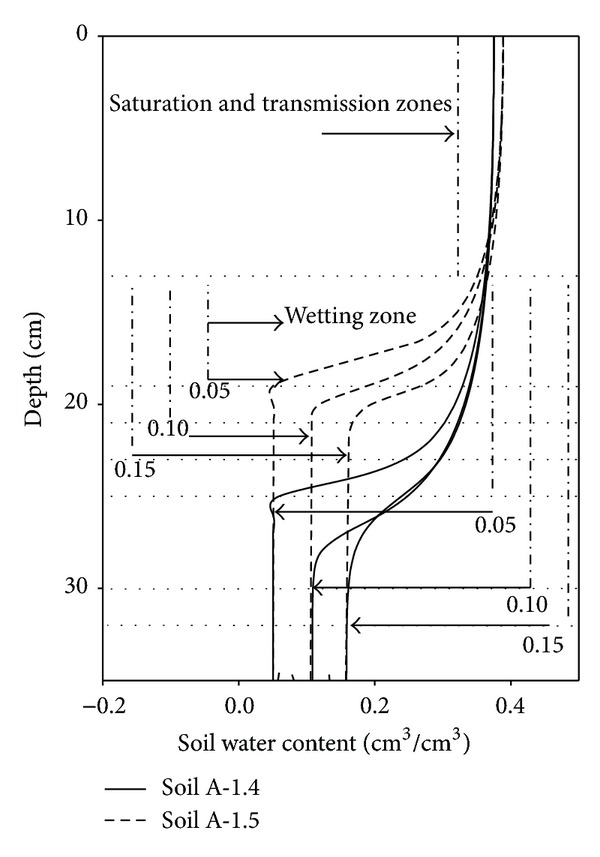
Simulated soil water content profiles after 30 min of infiltration in columns of Soil A with two different bulk densities (1.4 or 1.5 g/cm^3^) for different initial soil water contents (0.05, 0.10, and 0.15 cm^3^/cm^3^) and a ponding depth of 2.5 cm.

The initial soil water content significantly affected the wetting front depth, the thickness of the wetting zone and cumulative infiltration. For the *θ*_0_ values of 0.05, 0.1, and 0.15 cm^3^/cm^3^ the wetting front depths were 25, 30, and 32 cm, respectively, for Soil A with a bulk density of 1.4 g/cm^3^. For Soil A with a bulk density of 1.5 g/cm^3^, the corresponding wetting front depths were all reduced (19, 21 and 23 cm). The thickness of the wetting zone increased with increases in *θ*_0_ while the thickness of the saturation and transmission zones combined remained unchanged (Figure 10). The results all show that the wetting front advanced faster with a higher *θ*_0_. This may be due to the greater influence of gravity on water movement when *θ*_0_ was higher and closer to saturation [28].

For the *θ*_0_ values of 0.05, 0.1, and 0.15 cm^3^/cm^3^, cumulative infiltration was 73.5, 67.5, and 53.9 mm, respectively, for Soil A with a bulk density of 1.4 g/cm^3^, but when the bulk density was 1.5 g/cm^3^ the corresponding values were 56.7, 51.7, and 44.9 mm. Hence, the cumulative infiltration decreased as the *θ*_0_ increased. The phenomenon whereby increases in *θ*_0_ increased the thickness of the wetting zone but decreased cumulative infiltration was also noted [27]. The wetter the soil is initially, the smaller the matric suction will be and, as infiltration proceeds, the matric suction gradient will decrease. Thus, soil water gradients dominate the water movement for a relatively short time but reductions in the matric suction gradients lead to declines in soil infiltrability over time.

Although the initial soil water content (*θ*_0_) and ponded water depth (*ψ*_top_) both had some effect on the infiltration process, the degree to which they affected the infiltration process differed. For Soil A with a bulk density of 1.4 g/cm^3^, as the *θ*_0_ doubled from 0.05 to 0.1 cm^3^/cm^3^ and increased by 50% from 0.1 to 0.15 cm^3^/cm^3^, the wetting front depth increased by 24% and 9%, respectively. The corresponding decreases in cumulative infiltration were 8.0% and 20.2%. When Soil A had a bulk density of 1.5 g/cm^3^, the corresponding increases in the wetting front depths were 10.5% and 19.0% and the corresponding decreases in cumulative infiltration were 8.9% and 13.1%. These results indicated that the initial water content had notable effects on the wetting front depth and the cumulative infiltration. As reported in Section 4.2, the ponded water depth had a small effect on the soil water gradients and the thickness of the wetting zone, whereas the effects on these two parameters of the *θ*_0_ value were more notable. Thus, the infiltration process was more sensitive to variations in initial soil water content than to changes in the ponded water depth.

Soil surface sealing was induced by the physical breakdown of soil aggregates by drop impact and by compaction, as well as by physicochemical dispersion that was related to the soil ESP [29]. However, due to the high amounts of free salts in the soil, clay dispersion and swelling may also have been reduced due to increases in the electrical conductivity of the percolating water as it dissolved the salts and mixed with the soil solution. As a result, this may affect the applicability of the mathematical models, especially when the initial moisture content is varied.

## 5. Conclusions

Column experiments were conducted to examine the infiltration process in two sodic soils that were collected from a coastal reclamation area at two locations; Soil A was more recently reclaimed (2007) and was also saline, while Soil B was reclaimed in 1960. The effect of compaction was also studied by packing the soils at two different bulk densities.

A model was developed to analyze the infiltration process based on a finite difference method. The model was validated by comparing the simulated results with the measured data as well as with Hydrus-1D model simulation results. The model performed as well as Hydrus-1D in fitting the measured data.

Compaction notably affected the water retention of Soil A and Soil B. The value of the parameters *α* and *n* decreased when compaction increased. For both soils, *K*_*s*_ values decreased significantly when compaction increased, but the *K*_*s*_ of Soil B was more sensitive to compaction than that of Soil A. The magnitude of *K*_*s*_ and the changes due to compaction were also affected by the salinity of the soil. This was due to the effect of soil solution electrolytes on clay swelling that could block soil pores and impede soil water movement. Therefore, *K*_*s*_ does not simply reflect soil physical quality due to this physical chemistry effect. In contrast, the *S* parameter of the modeled water retention curve could be used to indicate soil physical quality alone. The *S* parameter decreased notably when compaction increased. Thus, compaction affected the capillary fringe thickness, pore size distribution, saturated hydraulic conductivity, and soil physical quality.

As the ponded water depth was increased on the surface of Soil A, cumulative infiltration increased for a given infiltration time and the wetting front advancement was greater. However, the effects of the ponded water depth on the infiltration process in Soil A were relatively small.

The thickness of the wetting zone increased with increasing *θ*_0_ while the thickness of the saturation and transmission zones combined remained constant. Moreover, the wetting front advanced more rapidly when the *θ*_0_ value was higher, but cumulative infiltration decreased as the *θ*_0_ increased. Furthermore, the infiltration process was much more sensitive to changes in the *θ*_0_ than to changes in the ponded water depth.

The process of desalination improves the soil physical structure over time. But this study suggested that at some point in this process the soil physical structure can become more sensitive to compaction effects. This result is related to both the physical breakdown of soil aggregates and the clay dispersion as salinity decreases while ESP remains relatively high. On the other hand, Soil surface sealing was induced by the physical breakdown of soil aggregates by drop impact and by compaction, as well as by physicochemical dispersion that was related to the soil ESP. The lower clay contents will also limit the effects of the clay dispersion on the hydraulic properties. In such a soil, clay swelling can also become maximal inhibiting water flow. Treatment of the soil with calcium-based amendments would reduce these problems.

## Figures and Tables

**Table 1:** Some properties of the soils used in this study.

Soil	Soil organic matter (g/kg)	Clay content (%)	Sodium (Na) ion content (g/kg)	Exchangeable Na percentage (%)	EC_1 : 5_ (mS/cm)	Year of reclamation	Land use
Soil A	3.26 (±0.11)	8.5 (±0.59)	1.60 (±0.43)	68.9 (±3.36)	5.98 (±1.05)	2007	None
Soil B	15.27 (±0.57)	10.7 (±1.01)	0.10 (±0.09)	16.8 (±1.44)	0.36 (±0.22)	1960	Chinese cabbage

EC_1 : 5_: soil electrical conductivity of a 1 : 5 soil to water extract; data represented by mean ± standard error (SE).

**Table 2:** Soil hydraulic parameters and conditions in the infiltration experiments.

Soil	Bulk density (g/cm^3^)	Ponding head (cm)	*θ*_*r*_ (cm^3^/cm^3^)	*θ*_*s*_ (cm^3^/cm^3^)	*α* (cm^−1^)	*n*	*K*_*s*_ (cm/min)	*θ*_0_ (cm^3^/cm^3^)	*S*
Soil A	1.4	2.5	0.001	0.3751	0.0050	2.0904	0.025	0.05	0.1534
		2.8							
	1.5	2.6	0.001	0.3889	0.0046	1.6157	0.020		0.1059
		3.9							

Soil B	1.4	2.7	0.001	0.4216	0.0056	2.4136	0.004	0.02	0.2093
		3.0							
	1.5	2.8	0.001	0.3848	0.0027	2.1267	0.002		0.1612
		2.7							

Note: *θ*_*r*_, *θ*_res_, and *θ*_sat⁡_ are the initial, residual, and saturated soil water contents (cm^3^/cm^3^); *α* (cm^−1^) and *n* (dimensionless) are unsaturated soil parameters; *K*_*s*_ is the saturated hydraulic conductivity (cm/min); *S* is a soil physical parameter.
